# Happy New Year

**Published:** 1879-01

**Authors:** 


### Editorial.

### "We wish you a Happy New Year."

We most sincerely and truly wish our readers a happy and prosperous New Year, and of course, presume upon their return of "Thank you, wish you the same." Under ordinary circumstances that would be quite adequate return, but it is unnecessary to say, that resumption of specie payment changes the situation.

Our publishers call early, January 1st, and in an earnest tone announce their good wishes for the New Year. JOURNAL replies, thank you, wish you the same; No says Mr. Pub. we now require specie, we have taken your excuses, promises, good wishes—your *paper*, until we can't use it any longer, and are obliged to demand the legal currency of 1879. JOURNAL looks a little disturbed in the countenance, and slowly replies, My dear sir, specie payment is attended with embarrassment and delay. Many of our subscribers don't pay in specie or any thing else. "*Stop*," says Mr. Publisher, I have had all I want of that—I have taken it of *some* of your subscribers for many years, and never a cent of anything else, and now if they read the JOURNAL as regularly as they receive it, post paid, and think as much of it as they seem to from the length of time since first subscription, they must be by this time *gentlemen* at least, and no *gentleman* will receive your JOURNAL a life-time and not profit enough by it, to be ready to report the payment of his subscription bill about January 1st, 1879, when every thing is to be paid in gold." JOURNAL rises up calmly and pacing the room thoughtfully, inquires of itself in an undertone, so as not to be understood, will our delinquent subscribers pay their bills? is there any possible way to reach their sensibilities? they have always been good friends of ours, and we have acquired a profound respect for them, and the question is now in point, can we rely upon them to satisfy the just claims of our publishers? No of course not, we have tried it too long, but we *will* try again, and if we fail, God have mercy on us all, as Abernethy said when first entering his lecture room and looking round upon the members of his class; and especially, God have mercy upon BUFFALO MEDICAL AND SURGICAL JOURNAL, and put it in the hearts of every delinquent subscriber to *pay his bill.*

